# Effect of Lutein and Antioxidant Supplementation on VEGF Expression, MMP-2 Activity, and Ultrastructural Alterations in Apolipoprotein E-Deficient Mouse

**DOI:** 10.1155/2013/213505

**Published:** 2013-04-30

**Authors:** Patricia Fernández-Robredo, Luis M. Sádaba, Angel Salinas-Alamán, Sergio Recalde, José A. Rodríguez, Alfredo García-Layana

**Affiliations:** ^1^Experimental Ophthalmology Laboratory, Clínica Universidad de Navarra, School of Medicine, University of Navarra, ES-31008 Pamplona, Spain; ^2^ Department of Ophthalmology, Clínica Universidad de Navarra, School of Medicine, University of Navarra, Pio XII 36, ES-31008 Pamplona, Spain; ^3^Atherothrombosis Research Laboratory, Division of Cardiovascular Sciences, Center for Applied Medical Research (CIMA), University of Navarra, ES-31008 Pamplona, Spain

## Abstract

Oxidative stress is involved in the pathogenesis of several diseases such as atherosclerosis and age-related macular degeneration (AMD). ApoE-deficient mice (apoE^−/−^) are a well-established model of genetic hypercholesterolemia and develop retinal alterations similar to those found in humans with AMD. Thus supplementation with lutein or multivitamin plus lutein and glutathione complex (MV) could prevent the onset of these alterations. ApoE^−/−^ mice (*n* = 40, 3 months old) were treated daily for 3 months with lutein (AE-LUT) or MV (two doses): AE-MV15 (15 mg/kg/day) and AE-MV50 (50 mg/kg/day) and were compared to controls with vehicle (AE-C). Wild-type mice (*n* = 10) were also used as control (WT-C). ApoE^−/−^ mice showed higher retinal lipid peroxidation and increased VEGF expression and MMP-2 activity, associated with ultrastructural alterations such as basal laminar deposits, vacuoles, and an increase in Bruch's membrane thickness. While lutein alone partially prevented the alterations observed in apoE^−/−^ mice, MV treatment substantially reduced VEGF levels and MMP-2 activity and ameliorated the retinal morphological alterations. These results suggest that oxidative stress in addition to an increased expression and activity of proangiogenic factors could participate in the onset or development of retinal alterations of apoE^−/−^ mice. Moreover, these changes could be prevented by efficient antioxidant treatments.

## 1. Introduction

Oxidative and nitrosative stress can induce alterations in DNA, proteins, and lipids, and extensive data suggest that oxidative damage may play a major causal role in a number of human diseases such as atherosclerosis, cancer, and cataracts as well as retinal pathologies such as age-related macular degeneration (AMD) [1, 2]. Currently, AMD is the most common cause of severe and irreversible blindness in Europe and the United States in people older than 65 years, and its prevalence is expected to increase as the population ages [3, 4]. The pathogenesis of AMD is unclear; however, several mechanisms influenced by genetic, systemic health, and environmental risk factors have been proposed. Numerous studies have also shown a relationship between cardiovascular disease and AMD, although others have not been able to verify this correlation. Dietary fat, in particular, cholesterol, is positively linked to increased incidence of coronary heart disease (CHD), and evidence suggests that abnormal lipid levels may contribute to the development of AMD, either directly or through the promotion of vascular disease [1, 2, 5]. 

Animal models attempting to recreate AMD through phototoxicity, senescence acceleration, candidate gene manipulation, and high-fat diets do not fully replicate the clinical, histologic, and angiographic features of the human condition, probably because of the multifactorial aspect of the disease [6]. The histopathology of early AMD reveals accumulation of specific lipid-rich deposits under the retinal pigment epithelium (RPE) [7]. Moreover, as it has been postulated on the hypothetical model of RPE oxidant injury, matrix metalloproteinases could participate in extracellular matrix (ECM) turnover in Bruch's membrane (BM) [8]. Degenerative changes of the RPE and photoreceptor cells are early events in AMD [9], and it has been demonstrated that apoE deficiency predisposes to ultrastructural changes in BM [10]. Apolipoprotein E-deficient mice (apoE^−/−^) develop spontaneous hypercholesterolemia in a few weeks [11] and also display morphological and ultrastructural alterations in RPE [10, 12, 13] similar to those in human AMD. Based on functional and structural analyses, the apoE^−/−^ mouse constitutes a valuable tool in elucidating the underlying mechanism of retinal degeneration [13]. 

Lutein and zeaxanthin are essential carotenoids that need to be obtained from certain vegetables, such as spinach, corn, pumpkin, and egg yolk [14]. They accumulate in the retina, where they play an important role in maintaining visual sensitivity and protecting against light-induced retinal damage [15, 16]. In the retina, lutein and zeaxanthin coexist with large amounts of polyunsaturated fatty acids that are highly susceptible to oxidation suggesting that antioxidants could prevent degenerative pathologies in which oxidative stress is of high importance such as AMD [17–20]. Our group previously showed an increase in oxidative processes related to the retinal morphological alterations observed in apoE^−/−^ mice and other models of hypercholesterolemia. Furthermore, we have reported the protective effect of antioxidants, such as vitamins C and E, lutein, egg yolk, and a multivitamin-mineral complex on retinal oxidative stress and hypercholesterolemia-derived ultrastructural alterations in apoE^−/−^ mice [21–24]. 

The aim of the present study was to investigate the effect of lutein and a multivitamin complex with lutein and glutathione on systemic and retinal biochemical and ultrastructural parameters in apoE^−/−^ mice. 

## 2. Material and Methods

### 2.1. Experimental Design

Ten 3-month-old male mice C57BL/6 and forty apoE^−/−^ mice were used for this study. Progenitor couples were obtained from “Center for Transgene Technology and Gene Therapy,” Flanders Interuniversity Institute for Biotechnology, Leuven (Belgium). All experimental procedures followed the Guidelines for the Use of Animals in Association for Research in Vision and Ophthalmology (ARVO) and were approved by the Animal Research Ethics Committee of the Universidad de Navarra. Animal welfare was applied during all experimental process and animals were euthanized by CO_2_ inhalation according to ethics guidelines. 

Animals were randomly divided into five experimental groups (*n* = 10), fed a standard rodent chow (9605/8, Harlan Teklad TRM, Madison, WI, USA) water ad libitum for 90 days, and housed in cages in a temperature-controlled room (20–22°C) with a 12-hour light/dark cycle. The five study groups were as follows: wild type (WT-C) and apoE^−/−^ (AE-C) receiving vehicle; apoE^−/−^ (AE-MV15) mice receiving 15 mg/kg/d of multivitamin-mineral, glutathione and lutein complex (providing 0.027 mg/kg/day of lutein) (composition in Table 1); apoE^−/−^ (AE-MV50) mice receiving 50 mg/kg/d of multivitamin-mineral, glutathione and lutein complex (providing 0.086 mg/kg/day of lutein); and apoE^−/−^ (AE-LUT) mice receiving 0.093 mg/kg/d lutein. Multivitamin treatment was administered according to the approved regimen for humans.

The treatments were emulsified in a mixture of water : soybean oil : Tween-80 (1 : 1 : 0.02; v : v : v) and administered daily by gastroesophageal cannula for 3 months (100 *μ*L). Purified lutein was kindly provided by Dr. Christine Gartner (Cognis, Germany), and multivitamin complex, Nutrof, was a kind gift by Laboratorios Thea (Barcelona, Spain). 

At the beginning and end of the treatment, eyes were examined by indirect fundoscopy with a 78-diopter lens and 0.5% cycloplegic eye drops. No retinal alterations were found in any group.

### 2.2. Lipid Plasma Analysis

Blood samples were collected after mice were killed and plasma obtained after separating the red blood cells by centrifugation (2,600 g, 10 min, 4°C) and were immediately frozen in liquid nitrogen and stored at −80°C. Concentrations of plasma total cholesterol (TC) and triglycerides (TG) were measured following the manufacturers' instructions (Sigma Chemical Co., St. Louis, MO), using a microplate ELISA reader and calculated from the linear range of standards.

### 2.3. Retinal and RPE-Choroid Homogenates Preparation

Immediately after blood collection, eyes were enucleated and transferred to a saline solution (pH 7.4). Retinas were rapidly dissected by making a small incision with a scalpel 1 mm behind the limbus and extending the incision through 360° using fine ophthalmic scissors. Anterior segment structures (cornea, iris, and lens) were removed. RPE-choroid samples were homogenized with a Teflon pestle in lysis buffer (RIPA buffer) and centrifuged for 20 minutes at 13,000 rpm at 4°C. Supernatant was collected and protein concentration was determined by Bradford assay with slight modification [21, 24]. 

### 2.4. Lipid Peroxidation in Plasma and Retinal Homogenates Based on Measurement of Thiobarbituric Acid Reactive Substances

Thiobarbituric acid reactive substances (TBARS) were measured in plasma and retinal homogenates as an index of oxidative stress, increasing the sensitivity by using a fluorometric modification of the method of Conti et al. as described [25]. Values were corrected by total protein content.

### 2.5. Measurement of Total Nitrites and Nitrates as an Indirect Indicator of NO Synthesis

Nitric oxide (NO) is oxidized rapidly in biological tissues, firstly to nitrites and secondly to nitrates. Determination of nitrites (NO_2_^−^) and nitrates (NO_3_^−^) is an indirect indicator of NO synthesis. Methodology employed for nitrites and nitrates measurement was adapted from Archer and Marzinzig [26, 27] with slight modifications. Briefly, samples were deproteinized with sulfosalicilic acid (25%), mixed with 5 *μ*L NaOH 1 M to arise pH 7,6, and centrifuged for 10 minutes (10,000 g). Standard curve was prepared with NaNO_3_ and ranged between 0 and 30 *μ*M. Fourteen mU of nitrate reductase (NADPH 40 *μ*M and FAD 1 *μ*M in Tris 20 mM; pH 7,6) were added to 10 *μ*L of supernatant. Samples were developed by adding diaminonaphthalene (DAN) 0.1 mg/mL and reaction was stopped with NaOH 2.8 mM. Fluorescence at 410 nm was read in a microplate reader (POLARstar Galaxy, BMG LABTECH GmbH) after excitation at 380 nm. 

### 2.6. Western Blotting for Vascular Endothelial Growth Factor (VEGF)

Equal amounts of RPE-choroid homogenates (5 *μ*g) were mixed with Laemmli buffer (62.5 mM Tris-HCl, pH 6.8; 2% SDS; 10% glycerol; 0.1% bromophenol blue) and boiled for 5 min. Samples were separated on 12% SDS-PAGE gels and transferred to a nitrocellulose membrane. After blocking with 5% skimmed milk (w/v), 0.1% Tween-20 (w/v) in TBS for 1 hour at room temperature, membranes were exposed to the primary antibody (0.2 *μ*g/*μ*L, monoclonal anti-VEGF, sc7269, Santa Cruz Biotechnology Inc., Santa Cruz, CA) at room temperature for 1 hour followed by incubation with a horseradish peroxidase-conjugated goat antimouse antibody (sc2005; 0.4 *μ*g/*μ*L, Santa Cruz Biotechnology Inc.). Signals were detected with an enhanced chemoluminescence (ECL) kit (ECL western blotting detection kit, GE Healthcare, Fairfield, CT) and exposure to autoradiographic film (Hyperfilm ECL, GE Healthcare). The relative intensities of the immunoreactive bands were analyzed with Quantity One software (version 4.2.2, Bio-Rad Laboratories, Hercules, CA). The loading was verified by Ponceau S red, and the same blot was stripped and reblotted with an anti-*β*-actin monoclonal antibody (Sigma-Aldrich) to normalize the VEGF level.

### 2.7. Gelatin Zymography Assay for Matrix Metalloproteinase-2 (MMP-2) Activity

MMP-2 activity was quantified by gelatin zymography on RPE-choroid homogenates [28]. Eight *μ*g of total protein from homogenate supernatants were mixed with nonreducing sample buffer (62.5 mM Tris-HCl, pH 6.8; 10% glycerol; 0.1% bromophenol blue) and electrophoresed directly on 9% SDS-polyacrylamide gels (SDS-PAGE) containing 0.1% gelatin (w/v). After electrophoresis, gels were washed 4 times for 20 minutes at room temperature in a 2.5% (v/v) Triton X-100 solution to remove excess of SDS, transferred to a solution (zymogram development buffer, Bio-Rad), and incubated for at least 18 hr at 37°C. Protein fixation was developed by incubating gels for 15 minutes with 50% methanol/7% acetic acid and then washing for 30 minutes (6 times of 5 minutes each) with distilled water. After that, gels were stained for 1 hour with GelCode Blue Stain Reagent (Pierce, Rockford, USA) counterstained with distilled water and then analyzed with Quantity One software (version 4.2.2, Bio-Rad) after densitometric scanning of the gels. The active MMP-2/(active MMP-2 + proMMP-2) intensity ratio was designated as the MMP-2 activation ratio. Each zymography assay was repeated at least three times to ensure accuracy.

### 2.8. Electron Microscopy

Three or four eyes from each group were processed for histological examination. The whole enucleated murine eyes were fixed in 2.5% glutaraldehyde, 0.1 mol/L cacodylate, 0.2 mol/L PBS. The posterior pole was dissected as described above and postfixed in 1% osmium tetroxide, stained with 1% uranyl acetate, and embedded in Epon Araldite resin. One-micrometer sections were cut with an ultramicrotome, stained with 2% toluidine blue O, and examined under a light microscope to determine the areas of interest. Thin sections (approximately 50–90 nm) were cut, collected on copper grids, and stained with 4% uranyl acetate and lead citrate. Subsequently, three sections from each animal were evaluated by transmission electron microscopy (TEM) (EM10, Carl Zeiss, Thornwood, NY) and photographed for posterior analysis. For semiquantitative scoring, 2-3 representative high-power micrographs were made of each low-power section. The high-power micrographs were graded by two independent examiners unaware of the experimental procedures. The variables evaluated were as follows: frequency of BLamD, BM thickness, presence of vacuoles in RPE, presence of vacuoles and lucent areas in BM, and presence of deposits of amorphous material in the RPE. BM thickness was directly measured in three different standardized locations in each image and averaged to provide a mean score for that micrograph. The mean of high-power micrographs was used to assign an average BM thickness for an individual specimen (*μ*m). The remaining variables were graded following previous score grading method [24, 29] with slight modification: absence (−), any (+), moderate (++), and severe presence (+++). In addition, the frequency of BLamD was classified as follows. “Any BLamD” was defined as the presence of any discrete focal nodule of homogenous material of intermediate electron density between the RPE cell membrane and BM in at least one micrograph. “Severe BLamD” was defined as the presence, in at least three micrographs, of the following: continuous BLamD extending under two or more cells, deposit thickness equal to or greater than 20% of RPE cell cross-sectional thickness.

### 2.9. Statistical Analysis

Values are reported throughout as the mean ± the standard error of the mean (SEM). Statistical significance for biochemical parameters and BM thickness was determined applying analysis of variance (ANOVA) or Kruskal Wallis test to assess differences among groups. After a significant ANOVA or Kruskal Wallis, comparisons between groups were made with Bonferroni posthoc or Mann Whitney test, respectively. Statistical significance was accepted at the 95% confidence level (*P* < 0.05), and analysis was performed by using the computer program SPSS (v. 15.0, SPSS Inc. Chicago, USA).

## 3. Results

### 3.1. ApoE^−/−^ Mouse Weight and Lipid Profile

Table 2 summarizes the general characteristics of the different animal groups. AE-C weight was higher than WT-C weight (*P* < 0.05), and apoE^−/−^ phenotype was confirmed by measuring plasma TC. Plasma TC and TG in the AE-C were significantly higher (*P* < 0.001 and *P* < 0.05, resp.) than in WT-C mice (Table 2). 

All mice had similar weight evolution during treatment, and treatments did not modify mice weight. Also, diet supplementation with nutritional supplement or lutein did not modify TC in apoE^−/−^ mice, showing that the effects observed in this study were independent of cholesterol concentration modifications. Nutritional supplementation in diet, in addition to not increasing plasma TC concentration, induces a decrease in TG concentration in apoE^−/−^ mice (*P* < 0.05). Lutein supplementation did not significantly modify TG levels (Table 2).

### 3.2. Plasma and Retinal Lipid Peroxidation

AE-C animals showed an increase in plasma and retinal lipid peroxidation (*P* < 0.01), as assessed by TBARS production, compared with WT-C (Table 3). 

Both doses of nutritional complex (MV) induced a reduction in systemic and retinal lipid peroxidation (*P* < 0.05 and *P* < 0.01) to values similar to control group (Table 3). AE-LUT animals did not show statistical differences regarding lipid peroxidation when compared to AE-C group (Table 3).

### 3.3. NO in Retina

Retinal NO synthesis was lower in AE-C compared with WT-C animals (*P* < 0.01, Table 3). All treatments administered were able to significantly (*P* < 0.05, Table 3) reduce NO synthesis compared to AE-C group.

### 3.4. VEGF Expression in ApoE^−/−^ Mouse and the Effect of Supplementation

**Figure 1:**
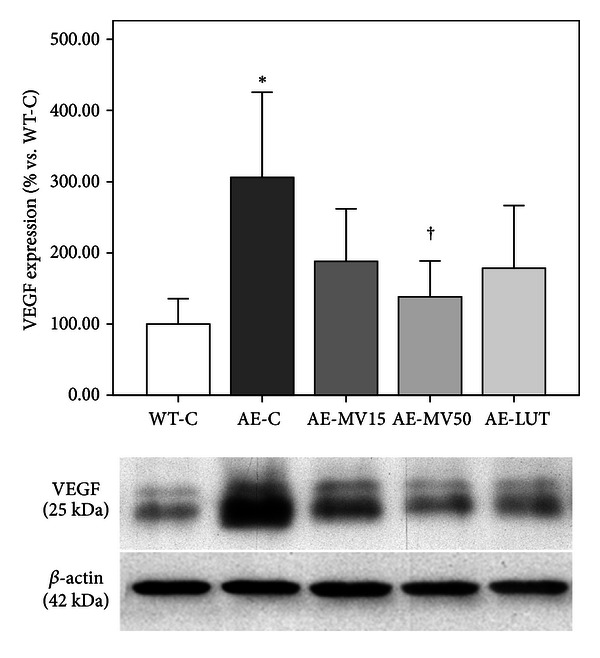
Changes in VEGF protein expression in RPE-choroid homogenates, reported as percentage increase with respect to untreated wt mice. Augmented VEGF protein in apoE^−/−^, assessed by western blot (densitometric analysis and representative blot showing the 25 kDa VEGF monomer), is reduced in apoE^−/−^ MV-50. *β*-actin was used as load control. Differences from WT-C are marked as *P < 0.05 and from AE-C as ^†^ *P* < 0.05 (*n* = 6-7).

Western blot with anti-VEGF antibody revealed an almost 3-fold increase in the AE-C group in comparison to WT-C (*P* < 0.05, Figure 1). 

AE-MV50 mice showed VEGF protein levels similar to WT-C (*P* < 0.05 versus AE-C, Figure 1). AE-LUT and AE-MV15 had a marginal reduction when compared to AE-C animals, but this difference did not reach statistical significance. 

### 3.5. MMP-2 Activity in ApoE^−/−^ Mouse and the Effect of Supplementation

**Figure 2:**
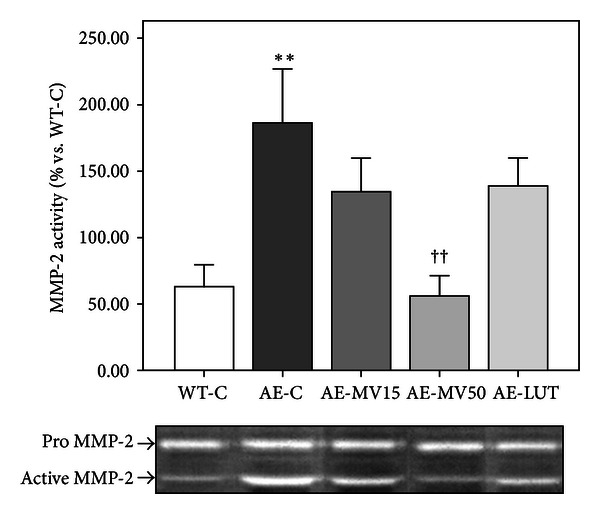
Zymogram analysis of MMP-2 activity in RPE-choroid, reported as percentage increase with respect to untreated wt mice. Eyecups homogenates of apoE^−/−^ mice showed an increase in gelatinase activity in comparison to WT-C mice. Mutivitamin treatment (AE-MV50) reduced MMP-2 activity back to control levels. AE-LUT and AE-MV15 groups exhibited a little but no significant reduction. Differences from WT-C are marked as **P < 0.01 and from AE-C as ^††^ *P* < 0.01 (*n* = 6-7).

Zymography analysis of total RPE-choroid homogenates showed that MMP-2 activity increased in AE-C groups when compared to WT-C tissues (*P* < 0.01, Figure 2). Gelatinase activity was significantly (*P* < 0.01) reduced in the AE-MV50 group compared with AE-C. AE-MV15 (*P* = 0.062) and AE-LUT (*P* = 0.072) animals did not show statistically significant MMP-2 activity modification, although a reduction was observed (Figure 2).

### 3.6. Ultrastructural Alterations in ApoE^−/−^ Mice and the Effect of Supplementation with Lutein and Multivitamin Complex

**Figure 3:**
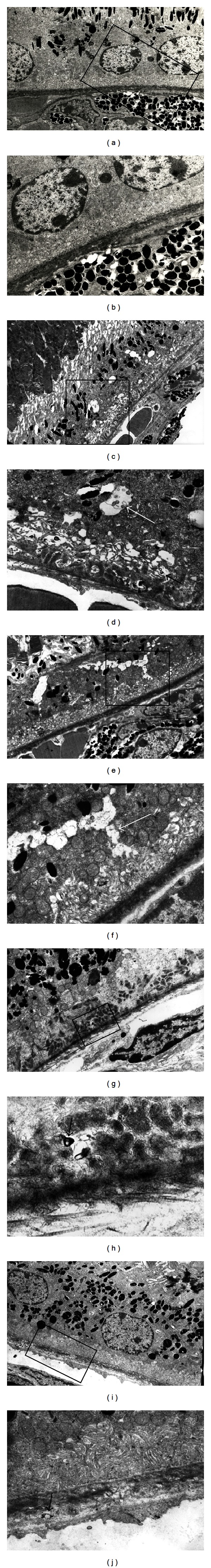
Transmission electron micrographs of the outer retina and choroid of WT-C (a, b) and AE-C (c–j) groups (*n* = 3-4). Photographs of wt mice reveal no accumulation of sub-RPE deposits, with normal RPE basal infoldings and BM ((a) ×2,250 and detail in (b) ×4,250). In contrast, apoE^−/−^ control mice show some ultrastructural alterations like vacuolization in RPE (white arrow), swelling of basal infoldings (black arrow), subRPE electrodense deposits (black asterisk) ((c) ×1,200 and detail in (d) ×5,250), and opening of intercellular junctions ((e) ×2,500 and detail in (f), ×5,250; white arrow). Also, electrodense material, seeming BLamD (black asterisk) is observed in RPE ((g) ×2,500 and details in (h) ×10,000) combined with some amorphous electro-dense material (black arrow). BM thickening is evident compared with WT-C animals, and non-membrane-bound lucent vacuoles inside BM were present ((i) ×3,975 and (j) ×7,725; black arrow).

**Figure 4:**
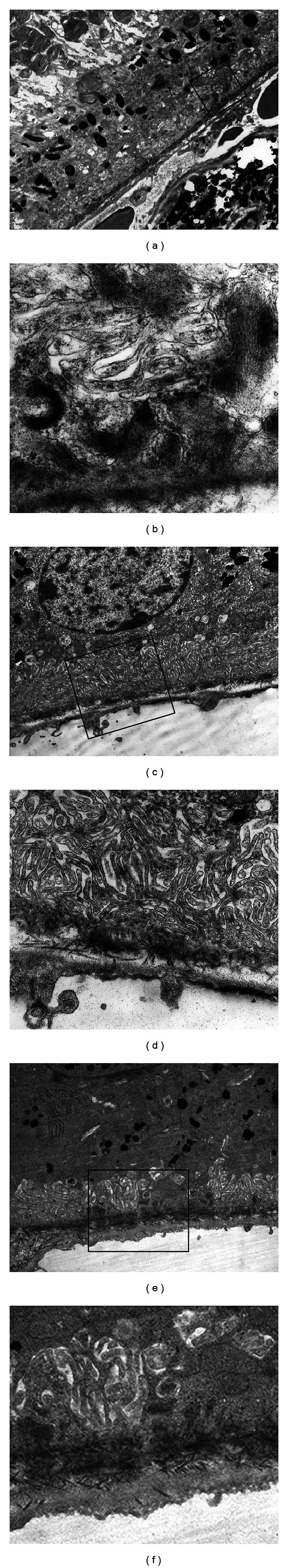
TEM from apoE^−/−^ animals supplemented with the different treatments. AE-MV15 ((a) ×2,500 and (b) ×10,000) and AE-LUT ((e) ×5,250 and (f) ×7,725) animals showed less confluent and more diffuse BLamD and less lucent areas in BM, whereas those alterations were almost absent in AE-MV50 mice ((c) ×5,250 and (d) ×7,725).

None of the eyes examined microscopically showed any type of drusen or neovascularization, but some alterations were observed in apoE^−/−^ mice (see below). ApoE^−/−^ mice showed basal laminar deposits, vacuoles, and an increase in BM thickness. While lutein alone partially prevented the alterations observed in apoE^−/−^ mice, MV treatment substantially ameliorated the retinal morphological alterations.  Figure 3 depicts photomicrographs of retinal cross sections from representative mice of WT-C and AE-C groups, Figure 4 shows representative images of the AE-treated groups, and Table 4 summarized the score grading for microscopic parameters analyzed.

WT-C mice showed normal RPE and BM features. RPE nuclei were rounded and displayed straight borders. The BM structure was conserved. TEM of the outer retina and choroid revealed no accumulation of sub-RPE deposits with normal basal infoldings, BM, and choriocapillaris (Figures 3(a) and 3(b)).

Compared with WT-C, AE-C mice showed ultrastructural changes (Figures 3(c)–3(j)). Low-power microphotographs revealed a disruption of cellular components and disorganized structure (Figures 3(c) and 3(e)). Also, eyes from AE-C mice had an increase in the number and size of empty and autophagocytic cytoplasmic vacuoles (Figure 3(d)) and, as previously reported [10, 13, 30, 31], basal laminar deposits-like (BLamD-like) structures located in the extracellular space between the basal lamina of the RPE and the inner collagenous layer of BM (Figures 3(c), 3(d), 3(g), and 3(h)). Moreover, most of RPE cells analyzed exhibited swelling of basal infoldings and opening of intercellular space junctions between RPE cells (Figures 3(e) and 3(f)). We also observed abnormal deposits of electrodense amorphous material in the subRPE space confined to a small area located to the RPE side of BM (Figures 3(d), 3(f), and 3(h)), as well as areas of increased thickness in BM (Figures 3(i) and 3(j)). The thickness of BM was statistically significantly higher in AE-C group than in WT-C (Table 4, *P* < 0.001). The BM of AE-C exhibited lucent non-membrane-bounded vacuoles dispersed through both collagenous layers (Figures 3(i) and 3(j)).

In contrast with the apoE^−/−^ untreated animals, mice fed with the MV complex exhibited less severe structural alterations in RPE and BM, with less swelling of basal infoldings and less cytoplasm vacuoles in the RPE and in BM. Mice supplemented with the low dose of multivitamin complex (AE-MV15; Figures 4(a) and 4(b)) and lutein (AE-LUT; Figures 4(e) and 4(f)) still showed moderate presence of BLamD in RPE, which were absent in AE-MV50 animals (Table 4, Figures 4(c) and 4(d)). BM thickening in all groups supplemented was less pronounced compared to AE-C animals. However, only in AE-MV50 group a statistically significant reduction was observed (Table 4, *P* < 0.05). 

## 4. Discussion

In this study we examined the utility of apoE^−/−^ mice as a model for AMD-like retinal degeneration and the effects of antioxidant treatments on the phenotypical and biochemical changes observed in this model. The most important findings are the increased VEGF expression and MMP-2 activity in the RPE-choroid of apoE^−/−^ mice and the corresponding decrease in VEGF levels and MMP-2 activity after high doses of a multivitamin complex with lutein and glutathione. In addition, multivitamin supplementation reduced systemic and retinal oxidation and ameliorated the pathological AMD-like changes in apoE^−/−^ mice. The fact that lutein and the multivitamin complex prevented biochemical and morphologic changes strongly suggests that hypercholesterolemia-derived oxidative stress is at least partly responsible for the alterations.

Based on biochemical and structural analyses, the apoE^−/−^ mouse is a valuable tool in elucidating the underlying mechanism of retinal degeneration. ApoE^−/−^ mice accrued marked increases in plasma TG and TC that were associated with increased systemic oxidative status. TG levels were reduced in animals supplemented with high-dose multivitamin complex, and this reduction may contribute, at least in part, to systemic oxidative stress decrease. We and others have reported evidence of systemic oxidative stress in apoE^−/−^ mice [22–24], and our group has previously demonstrated that systemic and retinal oxidative stress in apoE^−/−^ mice is reduced by dietary antioxidants present in egg yolk [23] and zeaxanthin [22]. Moreover, vitamins C and E were effective in reducing oxidative and nitrative stress in apoE^−/−^ mice [24] and in a model of porcine dietary hypercholesterolemia [21]. VEGF can be regulated by hypoxia, oxidative stress, and nitric oxide [32]. Matrix metalloproteinases (MMPs) are involved in extracellular matrix remodelling. Both VEGF and MMPs are regulated, at least in part, by oxidative stress [33, 34]; thus, it is relevant to study their activation and expression in pathologies where oxidative stress seems to play an important role. 

In the present work we observed a reduction in total NO production in the retinas of apoE^−/−^ mice compared to wild-type animals which could be a consequence of a reduced nNOS activity and, subsequently, reduced NO production [35]. In our study, the treatments groups showed NO reduction compared to apoE^−/−^ controls. It is possible that the decrease or increase in reduced glutathione (GSH) concentration (as a consequence of supplement administration) is able to alter the effects of NO (GSH forms adducts with NO: S-nitrosoglutathione) without affecting the expression of NOS [36]. It has been demonstrated that GSNO and NO protect neurons from hydroxyl radical-induced oxidative stress *in vivo* by terminating lipid peroxidation and augmenting the antioxidative potency of GSH, among other effects [37]. Therefore, restoring GSH levels can help NO to exert its beneficial effects, whereas the decrease in GSH levels could enhance the neurotoxic effects of free NO. Alterations in NO synthesis and bioavailability not only activate growth factors as VEGF but also induce physiological changes such as vasoconstriction and decreased choroidal flux. Increased oxidative stress in apoE^−/−^ mice could result in an excess of O_2_^•−^, which would react with NO to form ONOO^−^, decreasing NO bioavailability. However, the methodology employed in the present study did not permit us to evaluate NO bioavailability in genetic hypercholesterolemia. 

ApoE^−/−^ mice showed an increase in VEGF expression and MMP-2 activity in the RPE compared to wild-type-animals, probably produced in response to high oxidative stress environment. VEGF expression is regulated partly by oxidative stress or NO and hypoxia in RPE cells [38]. Our results agree with those obtained in apoE^−/−^ mice aortas showing an increase in gelatinase activity, VEGF, and VEGFR2 compared to controls [39, 40]. VEGF and MMPs can positively regulate each other [41]. An increase in VEGF expression in RPE has been previously reported by us; however, to our knowledge this study is the first showing an increased MMP-2 activity in posterior eyecups of apoE^−/−^ mice. It is possible that the augmented VEGF expression and MMP-2 activity in apoE^−/−^ mice could be derived, at least partly, from the increased oxidation observed in the retinal environment, where the presence of free radicals could stimulate cytokine or growth factors production. 

GSH and its related enzymes are part of the antioxidant defense against oxidative stress elevation. GSH depletion occurs in several forms of cell death, including in the retina [42, 43]. In *rd*1 mice it has been demonstrated that antioxidant supplementation (lutein, zeaxanthin, *α*-lipoic acid, and GSH) prevents photoreceptor apoptosis and DNA oxidative damage. When administered individually, none of the antioxidants produced a significant decrease in the number of apoptotic cells or DNA damage level in photoreceptors [44]. These results agree with ours in showing a decrease in retinal lipid peroxidation, VEGF, and MMP-2 activity in animals supplemented with the mixture of antioxidants with lutein and GSH but no significant modifications in animals supplemented with lutein alone. Our group demonstrated the decrease of superoxide anion as well as retinal lipid peroxidation by vitamins C and E oral administration in hypercholesterolemic porcine RPEs [21]. That result confirmed the association of retinal lipid peroxidation and changes in reactive oxygen species synthesis and could be the same mechanism in apoE^−/−^ mice and the multivitamin complex.

This is the first study showing a decrease in VEGF expression and MMP-2 activity in the retina after nutritional supplementation in the apoE-deficient mouse. According to the most recent review on lutein [45], there is only one study that demonstrated a reduction in VEGF after lutein supplementation where Izumi-Nagai et al. [46] showed a reduction of VEGF in RPE protein extracts from a murine model of choroidal neovascularization. Their experimental setting is completely different from our model; lutein was administered only during 6 days (3 days before laser photocoagulation and 3 days afterwards), while our treatment lasted for 3 months. Moreover, to the best of our knowledge, there is only one paper showing a decrease in VEGF expression in ARPE-19 cells after TNF-alpha stimulation [47].

To determine whether the biochemical changes are accompanied by retinal ultrastructural alterations, we performed limited electron microscopy analyses. In the future, extensive examination of several eye cross sections will render a more accurate depiction of eye-wide morphological changes. Nevertheless, our electron microscopy evaluation shows that apoE^−/−^ mice develop several ultrastructural alterations. BLamDs, defined as amorphous electron-dense material among the basal infoldings on the RPE side of BM, were present as previously reported [10, 22–24]. We observed ultrastructural changes in the BM of apoE^−/−^ similar to those reported by Dithmar et al. [10]. BLamD was more dispersed, and the sub-RPE layer contained far less electron-dense material in animals supplemented with multivitamin complex than in the controls or the untreated groups. The impact of dietary antioxidants on BLamD is consistent with reports that increased exposure to oxidation (e.g., inhalation of cigarette smoke and exposure to photooxidative stress) induces increased formation of BM deposits [29, 48]. Lipid peroxidation is likely one of many potential stimuli of injury, and it is possible that BLamD formation reflects a final common pathway of reparative processes shared by many cellular types in response to injury [49]. Our results strongly suggest that the reduced quantity of BLamD in the 6-month-old AE-MV50 retinas resulted from increased turnover or reduced formation of BLamD. Furthermore, we demonstrate an increase in BM thickness in apoE^−/−^ mice, that is, reduced by supplementation with antioxidants, lutein and GSH. The results obtained in this animal model agree with those showed in a porcine model of dietetic hypercholesterolemia [21] and with the observations in other models of retinal degeneration such as *rd*1 mice [44]. Our hypothesis is that the hypercholesterolemic status of apoE^−/−^ mice may have contributed significantly to the retinal abnormalities, probably mediated by an increase in oxidative stress. 

Moreover, this hypothesis is supported by the fact that those mice supplemented with multivitamin complex did not show these characteristic features. Therefore, these compounds could play a role in protection of oxidative stress-derived damage in the retina. This could be considered in preventive therapy for ocular degenerative pathologies, as a mean to improve life quality in patients. The reduction of VEGF expression and MMP-2 activity, accompanied by oxidative stress reduction, could be one of the mechanisms underlying the reduction in ultrastructural alterations. When lutein is combined with other antioxidant substances, beneficial effects are observed in hypercholesterolemia oxidative stress-derived changes. Although our study did not establish a causal relationship between phenotypical and biochemical findings, the latter can be associated with cytoplasmic and BM ultrastructural alterations. The increase in VEGF expression could be responsible for the increased permeability in choroid and BM, and MMP-2 activity could degrade BM, leading to the observed thickening and vacuolization. MMPs are enzymes involved in synthesis and degradation of extracellular matrix and have specific substrates such as collagen and elastin, both of them BM components. RPE cells are subsequently stimulated to increase synthesis of MMPs and other molecules responsible for ECM turnover. Increased matrix turnover is characterized by increased MMPs and decreased collagens [8]. As these morphological changes are ameliorated with supplementation with antioxidants, it is probable that oxidative stress increase is the initiating factor for the molecular and morphological changes in apoE^−/−^ mice.

## 5. Conclusions

This study provides data suggesting an evolving role for hypercholesterolemia in the development of retinal oxidative stress and the influence of dietary fat in ultrastructural RPE changes. Furthermore, VEGF and MMP-2 are responsible, at least in part, for some of the changes caused by hypercholesterolemia-derived oxidative stress. Additionally, supplementation with lutein, glutathione, and a complex of vitamins appears to be effective in reducing these retinal changes. However, further experimental, epidemiological and clinical studies are required to confirm these findings.

## Figures and Tables

**Table 1:** Daily dose per body weight of substances in the multivitamin-mineral complex.

	100 g	15 mg/kg/day (*μ*g)	50 mg/kg/day (*μ*g)
Vitamins			
Vitamin A	71.7 mg	0.32	1.08
*β*-carotene	0.4 g	1.8	6
Vitamin C (ascorbic acid)	10.7 g	48.15	160.5
Vitamin E (d-*α*-tocopherol)	1.8 g	8.1	27
Vitamin B_1_ (thiamin)	250.8 mg	1.13	3.78
Vitamin B_2_ (riboflavin)	286.6 mg	1.3	4.33
Vitamin B_3_ (niacin)	3.2 g	14.4	48
Vitamin B_6 _(pyridoxine)	358.3 mg	1.61	5.37
Vitamin B_9_ (folic acid)	35.8 mg	0.16	0.54
Vitamin B_12_ (cyanocobalamin)	179.2 *μ*g	0.00081	0.00269
Oligoelements			
Zinc (Zn)	1.3 g	5.85	19.5
Magnesium (Mg)	1.8 g	8.1	26.9
Manganese (Mn)	179.2 mg	0.81	2.69
Selenium (Se)	4.5 mg	0.02	0.066
Others			
Glutathione	179.2 mg	0.81	2.69
Lutein	179.2 mg	0.81	2.69

Data per 100 g of product are shown as mg and for daily dose in *μ*g. Calculation based on a 30 g mouse.

**Table 2:** Body weight, TC, and TG of the different animal groups at the time of sacrifice.

	Body weight	TC	TG
WT-C	28.3 ± 3.3	69.9 ± 9.5	83.2 ± 11.6
AE-C	35.6 ± 3.7*	624.7 ± 169.0***	123.4 ± 15.0*
AE-MV15	34.3 ± 2.8*	675.7 ± 142.6***	104.7 ± 21.1
AE-MV50	34.4 ± 2.4*	663.1 ± 161.4***	86.4 ± 30.7^†^
AE-LUT	35.6 ± 2.3*	675.7 ± 189.9***	137.6 ± 53.3

Data are expressed as g (body weight) and mg/dL (TC and TG) ± S.D. Statistically significant differences from WT-C are marked as *P < 0.05 and ***P < 0.001 and from AE-C as ^†^ *P* < 0.05. (*n* = 8–10).

**Table 3:** Plasma and retinal lipid peroxidation of the different animal groups at the time of sacrifice.

	TBARS plasma	TBARS retina	NO retina
WT-C	1.22 ± 0.06	3.02 ± 0.33	11.03 ± 1.18
AE-C	1.66 ± 0.10**	8.72 ± 0.78**	5.91 ± 0.85**
AE-MV15	1.32 ± 0.08^†^	4.77 ± 0.43^††^	2.13 ± 0.27^†^
AE-MV50	1.25 ± 0.11^†^	6.58 ± 0.67^†^	3.64 ± 0.48^†^
AE-LUT	1.57 ± 0.08	7.83 ± 0.36	2.21 ± 0.90^†^

Data are expressed as *μ*M malondialdehyde (MDA; TBARS plasma), nmol MDA/mg protein (TBARS retina), and nmol nitrates/mg protein (NO retina) media ± S.E.M. Statistically significant differences from WT-C are marked as *P < 0.05 and **P < 0.01 and from AE-C as ^†^ *P* < 0.05 and ^††^ *P* < 0.01 (*n* = 8–10).

**Table 4:** The effect of the different treatments on RPE and BM deposit severity, vacuolization, and BM thickness.

	WT-C	AE-C	AE-MV15	AE-MV50	AE-LUT
BM thickness	0.45 ± 0.03**	1.08 ± 0.05	0.78 ± 0.07	0.58 ± 0.04*	0.75 ± 0.06
Frequency of images with evidence of BLamD	−	+++	++	+	++
Presence of lucent areas in BM	−	++	+	−	+
Vacuolization in RPE	−	+++	+	+	+

Absence (−), any (+), moderate (++), and severe (+++) presence. BM thickness shown in *µ*m (mean ± SD). *P < 0.05 and **P < 0.001 versus AE-C (*n* = 3-4 for each group).

## Abbreviations

AE-C: ApoE^−/−^ mice treated with vehicle
AE-LUT: ApoE^−/−^ mice treated with lutein
AE-MV15: ApoE^−/−^ mice treated with MV (15 mg/kg/day)
AE-MV50: ApoE^−/−^ mice treated with MV (50 mg/kg/day)
AMD: Age-related macular degeneration
ANOVA: Analysis of variance
APOE^−/−^: ApoE-deficient mice
ARVO: Association for research in vision and ophthalmology
BLamD: Basal laminar deposits
BM: Bruch's membrane
CHD: Coronary heart disease
DAN: Diaminonaphthalene
ECL: Enhanced chemoluminescence
GSH: Reduced glutathione
GSNO: S-nitrosoglutathione
MMP-2: Matrix metalloproteinase-2
MV: Multivitamin complex
NADPH: Reduced nicotinamide adenine dinucleotide phosphate
nNOS: Neuronal NOS
NO: Nitric oxide
NO_2_^−^: Nitrites
NO_3_^−^: Nitrates
NOS: Nitric oxide synthase
O_2_^•−^: Superoxide anion
ONOO^−^: Peroxynitrite
RPE: Retinal pigment epithelium
TBARS: Thiobarbituric acid reactive substances
TC: Total cholesterol
TEM: Transmission electron microscopy
TG: Triglycerides
VEGF: Vascular endothelial growth factor
WT-C: Control mice.
